# Clinical applications of generative artificial intelligence in radiology: image translation, synthesis, and text generation

**DOI:** 10.1093/bjrai/ubae012

**Published:** 2024-08-13

**Authors:** Zhiqi Zhong, Xueqian Xie

**Affiliations:** Radiology Department, Shanghai General Hospital, Shanghai Jiao Tong University School of Medicine, Shanghai 200080, China; Radiology Department, Shanghai General Hospital, Shanghai Jiao Tong University School of Medicine, Shanghai 200080, China

**Keywords:** artificial intelligence, deep learning, radiology, diagnostic imaging

## Abstract

Generative artificial intelligence (AI) has enabled tasks in radiology, including tools for improving image quality. Recently, new hotspots have emerged, such as intra- or inter-modal image translation, task-specific image synthesis, and text generation. Advances in generative AI have facilitated the move towards low-dose, cost-effective, and high-quality radiological image acquisition. Large language models can aid radiologists by generating professional answers and facilitating patient-physician communications. However, radiologists must be aware of potential inaccuracies in the generated content and should only use such tools after rigorous validation of their performance.

## Introduction

Artificial intelligence (AI) techniques, represented by deep learning based on convolutional neural networks (CNN), have become common tools in radiology. While discriminative AI is commonly used, generative AI is an emerging branch aiming to produce new samples based on the training data. High-quality generated outputs not only produce valuable content but also enhance the performance of downstream discriminative models. Generative AI models were initially based on CNN and later on its variants, including the generative adversarial network (GAN) and Transformer architectures. Recently, various improvements have been proposed such as denoising diffusion probabilistic modelling (DDPM), relying on more complex and efficient algorithms to improve image generation.^1^

**Figure 1.**
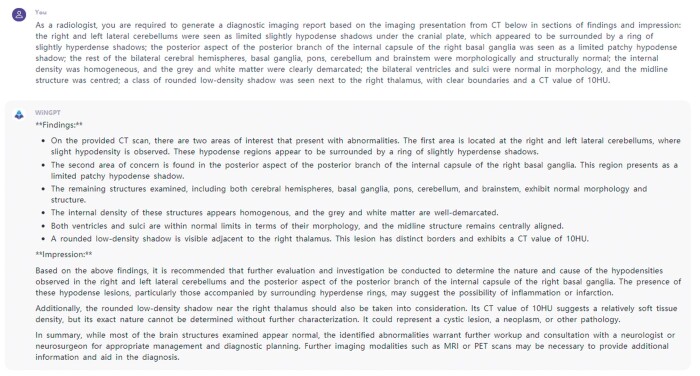
A large language model named WiNGPT successfully generated imaging findings and impressions from CT imaging descriptions as requested by free-text prompts. WiNGPT is publicly available at https://github.com/winninghealth/WiNGPT2.

**Figure 2.**
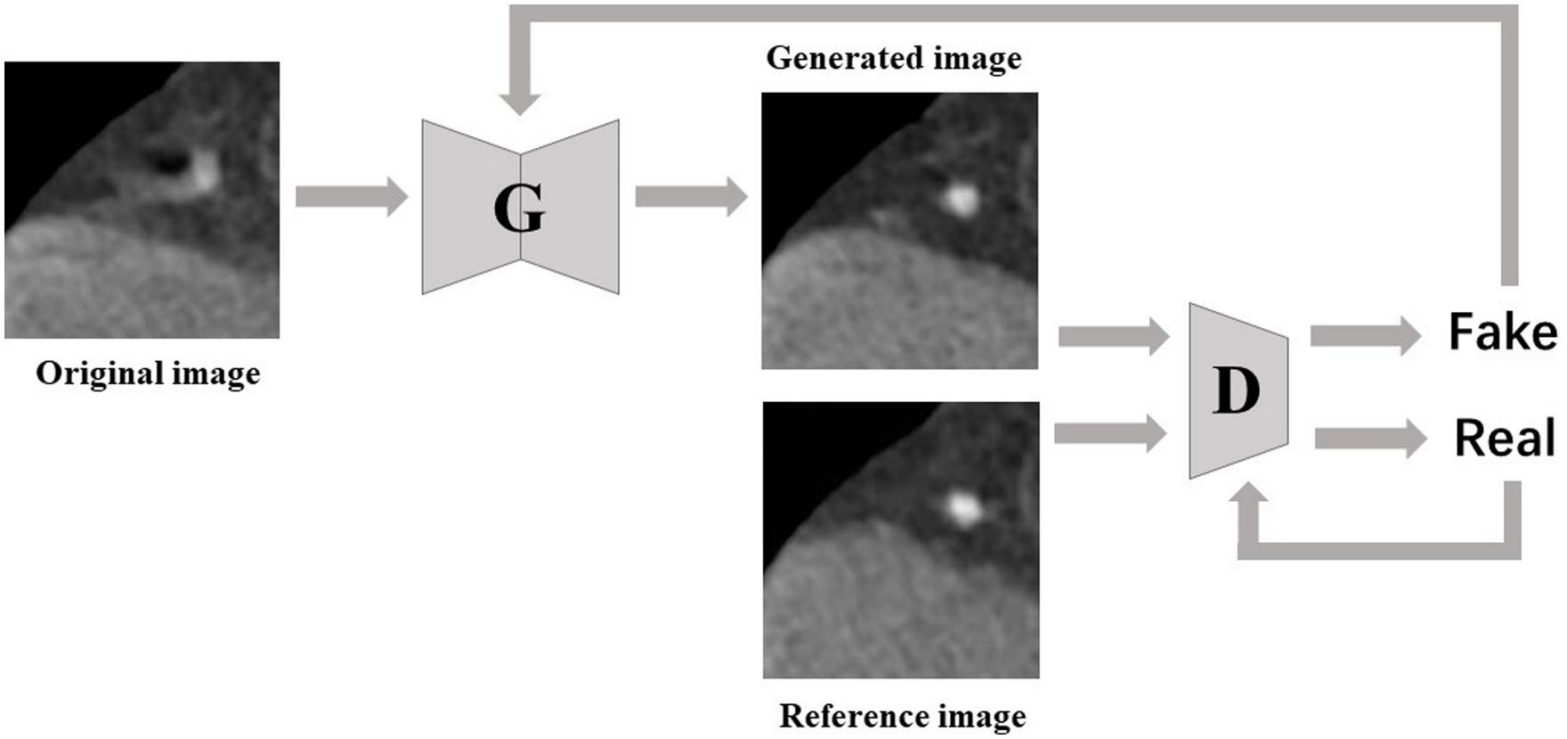
Working principle of Pix2Pix GAN: first, the original images are input into G (generator), and its output is later input into D (discriminator) together with the reference images. Through adversarial iteration between G and D, a dynamic equilibrium is finally achieved through competitive mode, where the generated image is close to the reference image and the discriminator cannot identify it as false, resulting in the final output.

**Figure 3.**
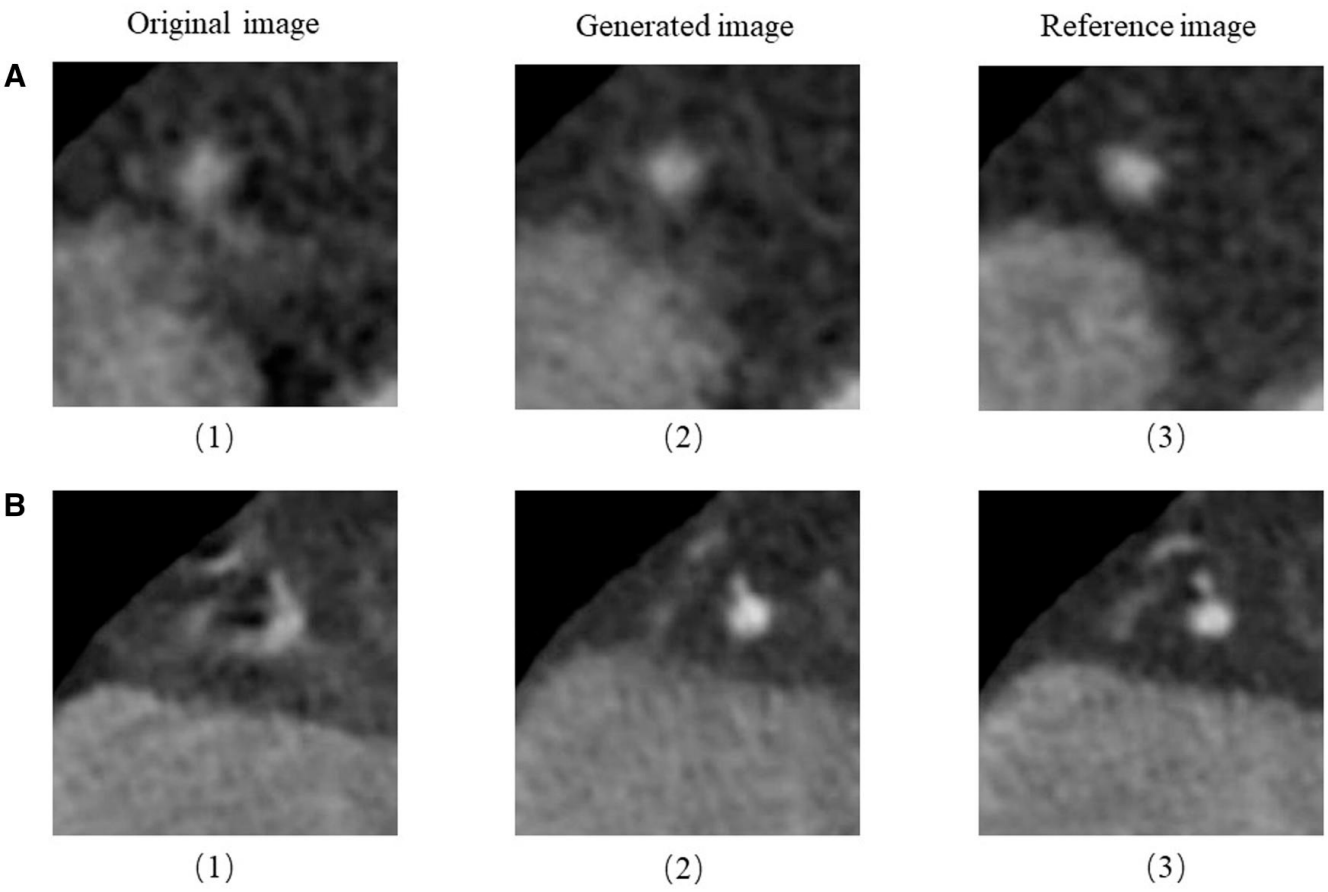
Two examples of image generation using Pix2Pix GAN to improve image quality by removing motion artefacts in coronary artery. (A) Single coronary artery with normal density. (B) Multiple coronary arteries with calcification. (1) The original image affected by motion artefacts. (2) High quality images generated by GAN. (3) An image obtained within the same cardiac cycle without artefacts and used as a reference.

In radiology, past generative AI research has focussed on improving image quality. A number of models for image quality enhancement have been released and validated. New models are being extended to cover more modalities and functionality.^1^ Recently, more scholars have begun to focus on intra- or inter-modal image translation and task-specific image synthesis. In addition, text generation tasks are also popular, such as generating expert responses, transforming radiology-related text, and generating radiological reports. Figure 1 shows an example of the AI’s ability to generate radiological text based on appropriate prompts. Figures 2 and 3 present an instance of the ability of generative models to improve image quality. This review summarizes the latest advances in generative AI in radiology, with focus on image translation, specific image synthesis, and text generation tasks.

## Image generation

Image generation can be categorized into image quality enhancement and image translation. The former is used for image reconstruction and post-reconstruction. Image reconstruction algorithms, such as AiCE and TrueFidelity, can be used to generate noise-reduced, high-quality images, although they are limited to vendor-specific devices.^2^ Recently, researchers improved image reconstruction algorithms, enabling additional features such as sparse-view CT reconstruction and a unified multimodal reconstruction framework.^3^^,^^4^

Techniques at the post-reconstruction level perform end-to-end operations, focussing mainly on denoising, artefact removal, and super-resolution. Several new models have been proposed. For example, a Transformer-based model was used to solve the limited receptive fields of CNNs, or to enable simultaneous local and global learning.^5^^,^^6^ In addition, GAN and diffusion models have emerged.^7^^,^^8^

Since work on image quality has been discussed in several reviews,^2^^,^^9^^,^^10^ this review focuses on image translation, with common models including U-Net, GAN, and DDPM.

### Image-to-image translation

Image-to-image translation enables the conversion of images from one form to another while preserving essential features. In radiology, image translation makes difficult-to-obtain images more accessible, thus saving time and technology costs. This section is about intra- or inter-modal image translation. Among these, the most extensively studied is the conversion between MRI sequences, followed by MRI to CT, and CT to other modalities.

### Image translation between MRI sequences

One common approach for converting images between MRI sequences is to generate difficult-to-obtain sequences from easy-to-obtain ones. Lee et al^11^ used Pix2Pix GAN to generate T2 weighted imaging (T2WI) from proton density-weighted imaging (PDWI) to save time. The Pix2Pix GAN employs a conditional GAN (CGAN)-based loss function, a U-Net-based generator, and a PatchGAN-based discriminator. The Pix2Pix GAN can produce pixel-level images, which resolves the blurring issue of the original GAN, but it requires each pixel to be labelled accordingly.^11^ Obtaining additional fat suppression sequences is a time-consuming process.^12^ Schlaeger et al^12^ used GAN to generate T2W-fatsat sequences with higher signal-to-noise ratios, reducing scanning time by about 40%. In the training of GANs, acquiring paired data is difficult. To tackle this challenge, CycleGAN circumvents the need for paired images by utilizing 2 sets of paired generators and discriminators.^12^ Chan et al^13^ subsequently used CycleGAN to generate diffusion weighted imaging (DWI) from fluid-attenuated inversion-recovery (FLAIR) sequences, thus overcoming the high cost of the DWI processing pipeline. The translation from DWI to FLAIR was inspired by the correlation of FLAIR’s imaging biomarkers with measures such as mean diffusivity and fractional anisotropy in DWI in brain tissue.^13^

Other innovations promote the translation between brain imaging sequences. For instance, conventional sequences do not reveal cerebral grey matter lesions.^14^ To address this issue, dual inversion recovery (DIR) and phase-sensitive inversion recovery (PSIR) sequences can be used, but they require a long acquisition time.^14^ Bouman et al^14^ used GAN to generate DIR and PSIR images from T1 weighted imaging (T1WI), T2WI, and FLAIR images, achieving a high level of comparability between the original and generated images. For perfusion imaging, Kossen et al^15^ used the Pix2Pix GAN with temporal components to generate expert-level perfusion parameter maps in an end-to-end manner. This framework modified the traditional 2D inputs into 3D containing time sequences. The framework introduced time components to improve performance by establishing a direct correlation between parameters and the correct order of time-intensity curves.^15^ Moreover, the exploitation of functional connectivity (FC) for blood oxygen level-dependent (BOLD) signalling between different brain regions helps to understand brain mechanisms.^16^ Oh et al^16^ used graph convolutional networks (GCN) as the backbone of GAN to facilitate FC generation, leveraging their ability to capture the fundamental topological features of FC.

Scholars have aimed to reduce the use of gadolinium contrast agents. Müller-Franzes et al^17^ used Pix2Pix GAN to recover contrast-enhanced images from low-contrast-enhanced images. Osman et al^18^ successfully eliminated the contrast agents by using dilated convolution in residual U-Net (ResUNet) to enhance the visibility of small anatomical features at different scales. To better display particular imaging features, image combinations can be used to generate high-contrast images. Touati et al^19^ employed a contrast learning strategy to represent paired data as similar and dissimilar pairs, allowing for better capture of anatomical structures. Since bidirectional models can learn from pairwise information, bidirectional synthetic loss is used to learn the best match between input features and target features. Thus, the images they generated retained more details of the vascular structure. Jacobs et al^20^ aimed at generating 4 MRI sequences, including T1WI, T2WI, PDWI, and T2W-FLAIR, from a single sequence based on a CGAN, with the result that the contrast of T2W-FLAIR was also improved. Using CGAN, researchers attempted end-to-end mapping of preprocessed data directly to target contrast, and methods based on physical signal models. The latter is more interpretable, with better T2W-FLAIR contrast, but still needs to address more artefacts.

### Image translation from MRI to CT

The image translation from MRI to CT enables excellent visualization of structures that are more clearly shown in CT. Vereecke et al^21^ validated the ability of BoneMRI software to convert MRI scans of sacroiliac joints to CT images and found it helpful in detecting lesions. Additionally, Arbabi et al^22^ used U-Net to bring the diagnostic accuracy of synthetic CT images of the knee to the level of real CT. Bird et al^23^ built a CycleGAN-based model using training data from multiple scanners to generate synthetic CT images of the pelvis, brain, and head and neck with high accuracy. Moreover, the training data for this software came from multiple scanners, making it clinically flexible, but the model still needs to be retrained based on new data to fit a wider range of sequences. However, the use of synthetic images for radiomics remains problematic. Yuan et al^24^ evaluated the capabilities of U-Net and CycleGAN and found that neither could effectively learn radiomics features of target images.

New deep learning architectures, such as diffusion model, may contribute to improve image translation. The DDPM model consists of 2 diffusion processes: forward diffusion, which gradually adds noise to the input and gradually removes its information; and reverse diffusion, which pushes back from the noisy image to the original.^25^ These processes capture complex high-dimensional distributions, allowing DDPM to produce higher-quality and more diverse images. Graf et al^25^ constructed the non-Markovian diffusion of denoising diffusion implicit model (DDIM) with a higher sampling rate. They used the U-Net, which combines convolutional self-attention and time-step embedding, to outperform the traditional Pix2Pix GAN. Furthermore, Pan et al^26^ improved DDPM, resulting in a reduction of the required time step by a factor of about 20. Another advancement is the Swin Transformer, which is based on the visual Transformer using sliding windows (shifted windows, Swin) to hierarchically divide the originally fixed-size sample blocks into differently sized blocks.^26^ These blocks are independent of each other and operate autonomously, significantly enhancing computational efficiency.

Furthermore, Table 1 presents other innovations in this area, such as improved imaging of sites with poor MRI contrast,^27^^,^^28^ limitations when using individual MRI sequence,^29^ feasibility of utilizing low-resolution CT as a reference,^30^ under-alignment of MRI and CT,^31^^,^^32^ ability to generalize models,^33^^,^^34^ and implementation of multimodal tasks.^35^^,^^36^

**Table 1.** Other innovations in image translation from MRI to CT.

Authors	Publication date	Issue	Details
Longuefosse et al.	July 2023	Improvement of poorly imaged body areas	Ultra-short echo time (UTE) lung MRI captures very rapid decay of the lung signal, but the quality is still inferior to CT. The use of GAN to synthesize CT quality from UTE MRI successfully removed artefacts and compensated for structures that would otherwise be missed.^27^
Kaushik et al.	August 2023	Improvement of poorly imaged body areas	Using U-Net architecture to translating the zero echo time (ZTE) MRI suitable for bone imaging to CT improved the bone value estimation problem in synthetic CT.^28^
Li et al.	March 2023	Limitations of a single sequence as input	An adaptive multi-sequence fusion network using multi-sequence MRI as input aggregated multi-sequence information from different regions by contextual correlation. A transformer-based cross-modal pixel-level fusion method improved the structural performance of bone and small organs.^29^
Florkow et al.	October 2023	Utilizing only low resolution CT as reference	A method of downsampling the training data to reduce noise levels and alignment errors was used, complemented by high-resolution data with the same anatomy to achieve the goal.^30^
Zhou et al.	November 2023	Under-alignment of MRI and CT	Two successive stages were used. The first was iterative refinement to achieve better alignment. Then there was a knowledge distillation phase that transferred knowledge from multiple teacher networks to student networks, aggregating knowledge to improve performance. This method had fewer illusions than CGAN and CycleGAN.^31^
Gu et al.	May 2023	Under-alignment of MRI and CT	A GAN approach with a three-loss objective function using uniform perception, CycleGAN and style loss was used to solve the structural misalignment between MRI and CT for more accurate image translation.^32^
Texier et al.	December 2023	Improvement of generalization	Using CycleGAN, an attempt was made to achieve that MRI obtained from any centre could be translated into accurate synthetic CT. The method improved the robustness even if there was little data from each centre.^33^
Simkó et al.	August 2023	Improvement of generalization	A ResNet architecture was used, combined with a preprocessing step of deconstructing the MR signal into PD, T1 and T2 maps to make the model robust to arbitrary contrast MRI inputs, thus improving generalization.^34^
Zhou et al.	June 2023	multimodal task	A cascade-based multimodal simultaneous generation network was proposed to fuse the high-dimensional features of multichannel data with an attention module. T1WI was first converted into contrast-enhanced T1WI, T1 fatsat Dixon reconstruction, and T2WI, and then the above synthetic sequences were fused with the initial T1 to generate CT.^35^
Ozbey et al.	June 2023	multimodal task	Simultaneous multi-contrast MRI and CT synthesis tasks with higher quality than GAN were achieved using SynDiff.^36^

Abbreviations: CGAN = conditional GAN; GAN = generative adversarial network.

### Image translation from CT to others

CT images are readily available in clinical practice and are suitable for image translation tasks. In radiotherapy planning, the limited soft tissue contrast of CT makes it difficult to distinguish tumours from normal tissue, but the additional use of MRI increases both time and cost. Li et al^37^ used ResUNet to convert CT scans to T1WI MRI, images, achieving fine results in most cases. The next application involved hyperpolarized gas MRI, a functional lung imaging that visualizes regional lung ventilation in a single breath, but requires specialized equipment. Given that CT ventilation imaging can detect regional ventilation, Astley et al^38^ used a V-Net-based framework to convert multiple inflated CT scans into images that correlated well with MRI ventilation scans. Moreover, non-contrast CT and FLAIR MRI sequence are commonly used for stroke follow-up.^39^ Gutierrez et al^39^ used CycleGAN to implement the inter-translation between non-contrast CT and FLAIR images, which is useful for simplifying imaging of stroke follow-up.

Table 2 shows recent tasks that have been performed to convert between CT images to improve safety or reduce costs, including interconversions between enhanced and non-enhanced CT,^40^^,^^41^ monoenergetic and dual-energy CT,^42^^,^^43^ diagnostic and radiotherapy planning CT,^44^ and filtered back projection (FBP) and iterative reconstruction (IR) images.^45^

**Table 2.** Recent tasks of image translation between CT images.

Authors	Publication date	Translation type	Details
Lyu et al.	November 2023	Non-contrast CT to contrast CT	A GAN-based architecture was used. It had good accuracy for the diagnosis of vascular diseases. However, errors occurred in the enhanced region. Also, arteries constitute only a small portion of the body and better methods are needed to enhance the mechanism of attention to vascular structures.^40^
Kalantar et al.	June 2023	Contrast CT to non-contrast CT	A 3D CycleGAN was used, which facilitated the creation of CT databases with more homogenised contrast, and downstream model development.^41^
Jeong et al.	September 2023	Single-energy CT to dual-energy CT	Pix2PixHD and CycleGAN were used to perform translation from 70 KV images to 50 KV images, iodograms, and virtual unenhanced images. Pix2PixHD is a modification of the Pix2Pix GAN that allowed stepwise conversion of high-resolution images.^42^
Sheikhi et al.	November 2023	Single-energy CT to dual-energy CT	120 KV image was taken as input and translated into a dual-energy image of the kidney region at 80 KV and 135 KV using Pix2Pix-UNet-GAN, which accurately predicted the stone type.^43^
Hooshangnejad et al.	June 2023	Diagnostic CT to planning CT	Diagnostic CT is used to depict tumours, but additional planning CT still need to be acquired. Shorter treatment initiation times are associated with improved survival, and 3D Pix2Pix were used because the difference between them was clearer in a large field of view.^44^
Lee et al.	September 2023	FBP and IR images	Inter-translation between FBP and IR images was achieved using CNN. Reconstructed images with different spatial resolutions and noise textures are a source of variability in radiomics studies. This study allowed the intensity and texture features of the tumour to show significant improvements in reproducibility.^45^

Abbreviations: CNN = convolutional neural networks; FBP = filtered back projection; GAN = generative adversarial network; IR = iterative reconstruction.

### Other image translation

Cone beam CT (CBCT) uses a tapered X-ray beam with more photon scattering and is inferior to conventional CT in visualizing soft tissue, in addition to other problems of fluctuating CT values and limited field of view. In order to convert CBCT to CT and improve image quality, Liu et al^46^ used the U-Net architecture to reduce artefacts and obtain more accurate CT values. Although it cannot use unpaired data like CycleGAN, it can save computational resources and training time.

CycleGAN, which is commonly used in generative AI, cannot guarantee structural consistency between CBCT and CT. Therefore, unsupervised models are still necessary when datasets are scarce.^47^ As CBCT can be considered a distorted input, it is possible to focus on only some of the elements. Inspired by this idea, Szmul et al^47^ applied a global residual learning approach incorporating structural consistency to facilitate anatomical soundness in synthetic images. Meanwhile, the method incorporated a novel intelligent data selection process that efficiently combined data from different cohorts to facilitate training. Table 3 lists other recent ideas^48–54^ to address the shortcomings of CycleGAN.

**Table 3.** Recent ideas to address the shortcomings of CycleGAN in image translation from CBCT to CT.

Authors	Publication date	Network architecture	Details
Liu et al.	September 2023	CycleGAN	A sequence-aware contrast generation network was built to improve image quality by exploiting the complementarity of information, using contrast learning to replace the loss of cyclic consistency and to achieve the style of conversion to CT while preserving the anatomical structure of the input CBCT.^48^
Deng et al.	May 2023	CycleGAN	The backbone structure of the CycleGAN generator was redesigned by adding a secondary chain to extract features from deeper levels. This dual chain CycleGAN allowed for more comprehensive information.^49^
Jihong et al.	April 2023	CycleGAN	Combining the traditional phantom correction method with CycleGAN, the CT values of CBCT images were first corrected and then used to train CycleGAN.^50^
Joseph et al.	March 2023	CGAN	Using a nested U-Net with a residual module as a generator for CGAN introduced the benefits of deep supervision into the model, providing additional supervision to the lower layers to enhance the overall accuracy.^51^
Wang et al.	January 2023	Registration GAN	Using a new approach that treated misaligned target images as noise labels was useful for image conversion tasks where pixel-level alignment cannot be achieved.^52^
Li et al.	October 2023	Frequency-guided DDPM (FGDM)	Inspired by the fact that the main spectral difference between CBCT and CT is located at the mid-frequency, this study designed FGDM to generate mid-frequency information by acquiring both high and low-frequency information.^53^
Gao et al.	August 2023	Swin Transformer	In addition to the direct translation of CBCT to CT, a new idea has recently been developed. A Swin transformer-based dual-domain network was used to estimate truncated regions in the sinusoidal map and the image domain to convert small-field-of-view CBCT obtained from low-dose, short-duration scans to a large-field-of-view mode.^54^

Abbreviations: CBCT = cone beam CT; CGAN = conditional GAN; GAN = generative adversarial network.

In addition to CBCT, a number of studies have been conducted with X-ray,^55–57^ nuclear medicine,^58–60^ ultrasound,^61^^,^^62^ and electrical impedance tomography (EIT),^63^^,^^64^ as listed in Table 4. Of these techniques, EIT has been used to generate low-resolution images of the lungs and other organs using noninvasive surface electrodes. However, techniques to address the low-resolution deficiencies of EIT are more computationally demanding, and generative AI has shown the potential to improve image quality.^63^^,^^64^

**Table 4.** Image translation studies on other modalities.

Authors	Publication date	Translation type	Details
Mori et al.	June 2023	Digital reconstructed radiography (DRR) to flat panel detector (FPD) images	Registration of FPD images to the DRR images takes some time, and different image qualities can make visual comparisons more difficult. This study used a U-Net-based 2D convolutional autoencoder that facilitated the throughput when visually comparing images of two different modalities in image-guided radiotherapy.^55^
Mochizuki et al.	November 2023	X-ray image to post bone suppression image	Adequate contrast of the tumour relative to the surrounding tissue is important. However, especially when bones overlap, tumour visibility is reduced after DRR images are matched to real-time X-ray images. This study used CycleGAN. There were bone intensity reduction difficulties stemming from the unrecognizable bones.^56^
Lee et al.	February 2023	CT to similar dual-energy chest radiograph	Dual-energy chest radiograph can increase the ability to differentiate anatomical tissues with two different energy of X-rays. However, noise and artefacts limit energy separation. This study used CycleGAN to successfully separate soft tissue from skeletal images by adding a loss of correlation coefficients to enforce structural similarity.^57^
Rajagopal et al.	July 2023	MRI to PET	Since MRI and PET images share a great deal of structural similarity, this study proposed a 3D ResUNet to translate contrast T1WI MRI to PET images. However, a limitation of output images being smoother than the reference was observed, due to the inherent flaws of CNN.^58^
Li et al.	February 2023	PET to CT	Although synthesizing CT from PET is a process of synthesizing information-rich images from information-poor images, this study used a GAN combined transformer and U-Net to successfully translate PET into CT to achieve PET self-base attenuation correction and reduce equipment cost and radiation.^59^
Du et al.	September 2023	Single photon emission computed tomography (SPECT) to attenuation-corrected image	CT-based attenuation correction improves the quality of SPECT, where CT imposes an additional radiation dose. This study used 3D CGAN in brain SPECT. The performance of the indirect method of generating attenuation maps first was verified to be superior.^60^
Chen et al.	July 2023	MRI to ultrasound	Intraoperative MRI provides a high-resolution view of anatomical structures in their entirety. This study compensated for the lack of intraoperative ultrasound data by using GAN to translate intraoperative ultrasound images directly from intraoperative MRI.^61^
Vukovic et al.	December 2023	Ultrasound to MRI	When lung and spinal cord surgeries are involved, traditional image-guidance techniques are limited in distance, but ultrasound can capture images more easily. This study used CycleGAN to translate real-time ultrasound into MRI that were more familiar to surgeons.^62^
Raza et al.	December 2023	EIT to CT	This study used CycleGAN to translate low-resolution EIT to high-resolution CT, where mutual information loss constraints were added to improve structural alignment between inputs and outputs.^63^
Wang et al.	July 2023	EIT to MRI	This study used CycleGAN to first generate a pseudo-high resolution EIT from the initial EIT, and then translated the latter to MRI, which was applied as an anatomical prior to the regularized EIT reconstruction.^64^

Abbreviations: EIT = electrical impedance tomography; GAN = generative adversarial network.

### Task-specific image synthesis

While the previous sections are fixed translation from one mode to another, the research involved in this section is based on the specific synthesis of practical problems, mainly including the synthesis of images for the purpose of expanding the dataset or to meet clinical needs, as well as the simultaneous synthesis of images from multimodal devices. Technological advances have made it possible to design specific models for particular tasks. Recently, Li et al^65^ proposed a noise modelling approach to generate low-dose CT data from conventional CT. They employed a 2-stage transfer learning training procedure for different noise styles to solve the problem that some models cannot adapt to different scanner noise styles. This model effectively separated valid content from noise in CT images and used a noise encoding network built into the generator to facilitate the model learning of different noise patterns. With the goal of simulating metal artefacts as realistically as possible, Li et al^66^ used GAN as the architecture and introduced a deep subnetwork to give the generator a strong capability to distinguish metal artefact features.

Furthermore, dynamic contrast-enhanced CT imaging necessitates the acquisition of images at different time points. But in certain instances, some images may be lost or rendered unusable.^67^ To prevent reacquisition, Raad et al^67^ proposed a CGAN-based architecture, where incomplete and complete image sequences are treated as random vectors, and the network generates samples in one vector as a condition for the other. For the case where truncated regions may appear in CT, Xie et al^68^ proposed a contextual attention-based GAN to complement the truncated regions by learning how to generate missing content by referring to the feature information of the known contexts. However, there were still some unsatisfactory results, mainly in the form of burrs and edge differences.

Generative AI has also been used to solve specific problems in MRI. Güllmar et al^69^ used StyleGAN, which introduced modifications to the generator to control the properties of the generated images, for image editing to analyse disease patterns and explore the characteristics of the disease stage over time. Besides, Alrumiah et al^70^ employed 2 GAN-based techniques, DCGAN and SingleGAN, to improve the imbalance between image data with and without tumours. DCGAN is a deep convolutional GAN architecture with CNN for both generator and discriminator to enhance the quality. SingleGAN is an unconditional fully convolutional GAN that learns from a single image and its internal distribution at different scales. Although DCGAN was trained with multiple images, it did not perform as well as SingleGAN.^70^

Functional MRI (fMRI) has seen new advancements with the introduction of the variational autoencoder (VAE)—GAN framework by Qiang et al.^71^ This framework addresses the limitations of insufficient high-dimensional data. The generator of the GAN produced data from the latent variables of the VAE, which helped the model to converge quickly. Unlike adversarial models, VAE aims to learn the true distribution of data, making it less susceptible to pattern collapse. However, the outputs can still be ambiguous. The VAE-GAN combines adversarial techniques to partially solve its limitation.^71^ To improve the simulation of temporal dynamics in fMRI and generate high-quality data, Wasserstein GAN was introduced. This method improves the training efficiency of GAN by solving the pattern collapse problem through estimating the distance between the distribution of data learned by the current model and the true distribution.

It is difficult to extract all the information of the disease from a single imaging modality, so nowadays, multimodal fusion images such as positron emission tomography (PET)-MRI are getting more attention.^72^ To tackle challenges such as high inference cost, Fan et al^72^ proposed a dual-path CT-MRI image fusion model based on a multi-axis gated multilayer perceptron. The framework used a combination of global and local paths to maintain the low complexity and achieved better performance than previous fusion models.

Finally, in the field of X-rays, Myong et al^73^ used a GAN-based model to generate chest radiographs with various pathological manifestations to balance the dataset, outperforming traditional methods. In PET, Karimipourfard et al^74^ extended the Pix2Pix GAN to generate images at different times based on the image of ^18^F-FDG at 60 minutes after injection. In ultrasound, Atri et al^75^ extended CGAN to successfully generate new images with limited data.

## Text generation

The main tasks of text generation are to produce professional answers, transform text information and generate reports. Commonly used models are Transformer-based large language models (LLMs).^1^ Compared to RNN, Transformer enables the transition from sequential to parallel processing, avoids gradient-related problems, and extracts features at a deeper level, making training extremely efficient. The popular Transformer-based LLMs are bidirectional encoder representation from transformers (BERT) and generative pretrained Transformer (GPT). BERT employs a bi-directional approach, utilizing information from both the front and back directions when predicting content. On the other hand, GPT uses an autoregressive approach, only using information from the left side of the text to predict the right side, similar to human writing. Thus, BERT is better at understanding textual content, while GPT focusses more on generating text.^76^

### Professional answers generation

ChatGPT is an LLM trained to provide detailed responses based on input prompts.^77^ Recently, there has been considerable interest in its ability to generate professional radiological responses. Currie et al^77^ tested the response of ChatGPT on the course exams for radiological undergraduate students in the initial 3 years. The results showed that ChatGPT performed well in the first 2 years, but struggled in the third year due to a lack of evidence and fabricated quotes in the provided answers. Kufel et al^78^ validated ChatGPT’s performance on multiple-choice questions in the Polish radiology professional examination, but the correct answers were only 52%. This study also indicated that ChatGPT was inadequate in handling complex problems, although this may be related to the fact that Polish was not the main language of the model. Meanwhile, Bhayana et al^79^ tested ChatGPT on English-language radiology image-free questions and achieved a 69% accuracy rate. Their results showed that the model performed well on problems related to clinical management, but poorly on physical problems. The success of ChatGPT in clinical management was attributed to the abundance of disease-specific clinical data available online. It is worth noting that even when generating incorrect answers, the model remained confident.^79^

Compared to the ChatGPT, GPT-4 provided remarkable improvements in both professional and academic benchmarks.^80^ In the study of Bhayana et al,^80^ GPT-4 passed the text-based radiology exam, scoring more than 10% above the passing threshold. Moreover, its improvement on higher-order problems supported performance in problems requiring advanced reasoning skills. However, GPT-4 remained confident in incorrect answers.^80^

Recently released by Google, Bard is similar in functionality to ChatGPT. However, it has the added ability to access the internet in real-time and uses a communication model called the language model for conversation applications.^81^ Patil et al^81^ compared ChatGPT, GPT-4, and Bard, and found that while Bard typically generated answers with longer word counts and was faster, possibly due to its current low usage, GPT performed better overall.

GPT-3.5-turbo is a new version adding the ability to fine-tune the model, and its common method of fine-tuning is to use LlamaIndex as the external data and OpenAI’s embedded dictionary model.^82^ Russe et al^82^ compared the ability of GPT-3.5-turbo and GPT-4 after fine-tuning in dental imaging. This study showed that the fine-tuned GPT-4 achieved 100% accuracy, although only 87.5% of these interpretations were adequate, which was still superior to the GPT-3.5-turbo and lower-ranking physicians.^82^

Additionally, several studies have targeted the LLM’s task in answering questions related to radiology. For instance, Scheschenja et al^83^ compared ChatGPT and GPT-4 in answering questions from patients before interventional operations. The results showed that both LLMs provided accurate answers, while GPT-4 was only slightly superior. Since GPT-4 was fee-based to obtain, there was no advantage in this task.^83^

For more clinically relevant tasks, some studies have explored the ability of LLM to generate imaging decisions. Gertz et al^84^ showed that the results generated by GPT-4 were highly consistent with the reference. However, due to the fact that the reference guidelines used by GPT-4 are unclear, larger studies as well as fine-tuning of GPT-4 are needed in the future.^84^ Moreover, Rao et al^85^ examined the capability of ChatGPT and GPT-4 for breast pain and cancer screening. Its accuracy compared with manual decision-making was broadly consistent. More specifically, ChatGPT tended to provide multiple imaging modes, whereas the question asked for only one. In addition, it could provide wrong rationale for incorrect decisions and fail to distinguish between similar but actually different imaging modalities. These limitations have been addressed in GPT-4.^85^

### Transforming text information

Text information transformation can be seen as a summary based on different needs, presented in a structured and simplified form. Structured reports are defined as reports with a predefined format that can reduce error rates, increase report completeness, reduce writing time, facilitate data extraction tasks.^86^ But some argue that structured reports may divert radiologists’ attention. Automated models may provide a solution by freeing up more time for radiologists. Adams et al^86^ verified GPT-4’s ability to transform free-text into structured reports in English and German for X-ray, CT, and MRI. Sasaki et al^87^ applied GPT-4 to interventional radiology reporting. However, they encountered problems with the model’s inherent ambiguity, which sometimes led to misinterpretation of certain terms and generated treatment plans not included in the original report, although mostly appropriate.

In addition, some new models were proposed. Fanni et al^88^ proposed a neurolinguistics model for question and answer, capable of operating in zero- or less-shot mode, which allows users to easily configure the model. This model underwent 2 iterations to structure the free-text COVID-19 chest CT report. Sugimoto et al^89^ used a 2-stage deep learning system. The first stage involved extracting language entities from the report, while the second stage involved extracting the relationships between them. It was demonstrated that long short-term memory (LSTM) outperformed BERT in entity extraction tasks, which in turn outperformed the former in relation extraction tasks. This is because local information is more vital in entity extraction, while relationship extraction tasks require more global information.

Radiology reports often contain complex terminology that can be difficult for patients to understand.^90^ To address this issue, LLM has been studied to simplify the report content. Li et al^90^ used GPT to simplify X-ray, CT, MRI, and ultrasound reports, reducing the readability level to below the sixth grade and shown human-like empathic responses from GPT. Furthermore, some studies have explored the use of new models. For instance, Jiang et al^91^ proposed a BERT-based pretrained Chinese medical language model to address the limitations of simplified Chinese reports.

### Generating reports

Recently, a few studies have attempted to allow LLM to generate reports based on image input. This is an image-text application that has been implemented in several scenarios. Zhang et al^92^ used BERT to extract linguistic entities and associations from unstructured radiology reports to train CNN to classify abnormal signs in chest radiograph, and later used rule-based natural language processing (NLP) tools to generate chest radiograph captions. The results suggested that the framework could provide physicians with priors by interpreting multiple signs of abnormality in chest radiograph.^92^ Also based on chest radiograph, studies have been done to achieve full report generation. Lee et al.^93^ compared KARA-CXR, a chest radiology assistant developed by KakaoBrain AI, with GPT-4 Version. This study showed that GPT who learned from publicly available information on the internet could not guarantee absolute medical expertise and therefore had inferior accuracy.^93^

ChatGPT is designed at the present to reject requests for interpretation of medical images in order to avoid its use as a substitute for doctors.^93^ Thus, the development of more specialized models is essential. In this regard, Yang et al^94^ used CNN and BERT to extract features from images and corresponding reports, respectively. Since the key information usually comes from the description of the anomaly, and such sentences are rare and diverse. This study proposed a multimodal alignment mechanism that aligns visual features and corresponding text to guide the model in storing anomalous content.^94^ Additionally, Selivanov et al^95^ similarly combined 2 language models based on image and textual attention, but included NegBio labels to add the appropriate topics to the beginning of the reports. These labels helped the model understand exactly where to generate text from and mitigated data imbalance by bringing the task closer to the classification task.^95^ However, the inherent localization of CNN when used as encoders may result in failure to extract visual information that matched the textual description.^95^ For this issue, Pan et al^96^ investigated the use of a dual-stream visual feature extractor with ResNet and Swin Transformer, and introduced self-supervised learning to allow the model to focus on imperceptible focal regions. In the report generation task, missing information due to undersampling of the dataset can be a challenge.^96^ To this end, a self-supervised learning method was employed to randomly mask the samples. This allowed the model to extract more detailed visual features during the recovery process. The introduction of self-supervised learning represents a significant breakthrough in this field.^96^

Other attempts have been made to improve radiology reports. When writing reports for CT scans, it is important to align multiple images and sentences. Existing frameworks have difficulty paying accurate attention to key areas. In response, Zhang et al^97^ proposed the weakly guided attention model with hierarchical interaction. This framework improved many-to-many matching of images and text by guiding spatial attention to lesion regions.

## Prospectives

Although text generation has solved many problems for radiologists, there are still new attempts. To address the problem of doctors spending a lot of time on computers rather than patients, the MEDALIGN dataset were proposed to enhance the capabilities of LLM in related tasks. As there is also much computer data in radiology, similar attempts can be made to reduce the burden on radiologists.^98^

In addition to improving task coverage, the direction of model development is important. Popular models can provide inspiration for model development. For example, LLMs pretrained on large datasets have changed the paradigm of NLP through zero-shot learning. Inspired by this, a new suggestive image segmentation model, Segment Anything, was established.^99^ Additionally, generative AI models require a huge amount of computation and encounter obstacles in data collection. Therefore, another direction is to simplify the models and save computational resources. Using the unpaired dataset of CycleGAN can reduce the model’s data requirement.^13^ Strategies incorporating contrastive learning can also enable the model to require fewer parameters and achieve higher computational efficiency.^19^ Meanwhile, new methods to reduce computational complexity have been attempted, such as incorporating attention-guided learning processes when processing unpaired data and proposing new loss functions.^39^ Not only GAN, but also DDPM is exploring ways to improve efficiency,^25^^,^^26^ such as changing the conventional way of predicting values and using non-Markov diffusion model, DDIM. It is foreseeable that more methods will be proposed to make generative AI models lighter and easier to implement.

## Conclusions

In radiology, the cutting edge of generative AI is centred on more creative aspects like image translation and specific synthesis, as well as generating relevant text. Intra- and inter-modal image translation, such as MRI and CT, has become practical can save resource costs. Image generation models have been able to perform more specific tasks, such as creating datasets in various specified styles. With regard to text generation, LLM can assist radiologists with their knowledge by generating professional answers, but their authenticity needs to be carefully judged. LLM's capabilities in report-related textual tasks have been extensively explored, and their potential to facilitate patient-physician communication has been demonstrated.

Generative AI is becoming increasingly advanced in radiology, with more practical functionalities and models emerging. As radiologists, it has become a trend to constantly keep up with it. It is foreseeable that more generative models will come into clinical practice. However, it is vital to realize that the content produced is likely to contain errors, and there may even be the possibility of manipulating the results by controlling artificially. Therefore, it is imperative that the development and application of generative models and their outputs should undergo strict regulation and rigorous validation.
